# The Effect of Rosemary and Oregano Extract Addition on Selected Quality Properties of Pork Pâtés During Cold Storage

**DOI:** 10.3390/molecules30224409

**Published:** 2025-11-14

**Authors:** Paulina Duma-Kocan, Mariusz Rudy, Marian Gil

**Affiliations:** Department of Agricultural Processing and Commodity Science, Institute of Food and Nutrition Technology, Faculty of Technology and Life Sciences, University of Rzeszow, Zelwerowicza 4, 35-601 Rzeszów, Poland; pduma@ur.edu.pl (P.D.-K.); mgil@ur.edu.pl (M.G.)

**Keywords:** rosemary extract, oregano extract, pork pâtés, physicochemical properties, textural profile, sensory properties, chemical composition, cold storage

## Abstract

This study aimed to evaluate the effect of rosemary and oregano extracts on the chemical composition, physicochemical parameters, and sensory characteristics of pork pâtés under cold storage for 1, 7, and 14 days. Five different experimental variants were developed: “k”—control sample (no extract added), “rr”—with 50% rosemary extract added, “rs”—with 100% rosemary extract added, “oo”—with 50% oregano extract added, and “os”—with 100% oregano extract added. The study showed that rosemary and oregano extracts did not cause significant changes in the basic chemical composition and pH. However, they significantly affected the oxidative stability, color characteristics, texture, and sensory acceptance of the pâtés. TBARS (lipid oxidation rate) values systematically increased during storage, with the lowest lipid oxidation rate observed in samples with rosemary extract. The extracts also limited the increase in oxidation-reduction potential compared to the control sample. Changes in texture parameters were also observed, but the additives significantly reduced their unfavorable character, particularly in terms of hardness and chewiness. Sensory evaluation results confirmed the positive impact of the extracts, particularly in terms of odor and taste, which were rated significantly higher than in the control sample. The conducted studies indicate that rosemary and oregano extracts may be a natural source of compounds with antioxidant properties and stabilize the quality of pork pâtés. Their use may provide an effective and consumer-acceptable alternative to synthetic preservatives, supporting the development of meat products aligned with the “clean label” trend.

## 1. Introduction

The contemporary food industry is increasingly challenged to satisfy consumers who demand products that are not only safe and flavorful but also perceived as natural and health-promoting. Consequently, there is growing interest in exploring plant-derived additives as alternatives to synthetic preservatives and antioxidants, with the dual goal of enhancing both the quality and the shelf-life of foodstuffs. Offal products, despite their status as valuable sources of nutrients, are particularly prone to biochemical and microbiological deterioration throughout storage. This vulnerability stems from their complex composition—a mixture of various saturated and unsaturated lipids, proteins, carbohydrates, vitamins and pigments—which creates multiple substrates for degradative reactions [1,2,3].

Oxidative processes mediated by reactive oxygen and nitrogen species play a central role in the deterioration of meat products’ quality. These reactions are promoted by environmental and processing factors such as light exposure, molecular oxygen, elevated temperatures, successive production steps and the presence of transition metals; the composition of animal feed likewise affects meat’s susceptibility to oxidation. Lipids and proteins are the primary targets: lipid peroxidation generates hydroperoxides that propagate secondary degradation pathways and give rise to undesirable volatile compounds (e.g., aldehydes, ketones, organic acids and alcohols) [4]. Moreover, physicochemical changes in proteins and amino acids during the oxidation process reduce their bioavailability, digestibility, solubility and proteolytic activity [5]. Oxidation can also negatively impact the appearance of the product by oxidizing myoglobin to oxymyoglobin and metmyoglobin and producing brown pigments. The above-mentioned reactions reduce the sensory and nutritional value of the product and negatively affect consumer satisfaction [6,7,8].

To prolong the shelf-life of meat products, it is common practice to incorporate antioxidants and/or antimicrobial agents. Conventional synthetic antioxidants frequently employed include BHT (butylated hydroxytoluene, E321), BHA (butylated hydroxyanisole, E320), TBHQ (tertiary butylhydroquinone, E319), gallates and citric acid [9,10]. However, these chemical additives have been associated with adverse health concerns—toxicity and potential carcinogenicity—and have consequently met with consumer resistance. Moreover, meat and meat products remain highly perishable due to contamination by foodborne pathogens, which can provoke color changes and the generation of volatile nitrogenous compounds such as ammonia, dimethylamine and trimethylamine [11,12]. In addition to synthetic antioxidants, nitrates and nitrites are among the most commonly used additives in the meat industry due to their multifunctional roles in color development, microbial control, and oxidative stability. These compounds contribute to the characteristic cured color of meat, inhibit the growth of Clostridium botulinum, and exhibit strong antioxidative effects, thereby enhancing the oxidative and sensory stability of meat products [13].

In response to health and perception issues linked to synthetic preservatives, there has been intensified effort to identify natural alternatives [14,15]. Research activity is focused on replacing chemical antioxidants with natural counterparts, particularly plant extracts that are rich in phenolic compounds and other bioactive constituents [1,8,16]. A substantial body of literature examines the potential of plant-derived substances—including many essential oils with strong antioxidant capacity—as substitutes for synthetic antioxidants in the meat industry. Many of these botanicals are classified as Generally Recognized as Safe (GRAS), and their application, either alone or in combination with other essential oils, adjunct ingredients or preservation technologies, can exert beneficial effects on meat quality and stability. The efficacy of such natural additives depends on factors including concentration, possible synergistic interactions and the extraction method used to obtain the active components [4,14,17].

Herbs belonging to the Lamiaceae family, such as oregano (*Origanum vulgare*) and rosemary (*Rosmarinus officinalis*), represent a rich source of biologically active phytochemicals, notably phenolic compounds such as carvacrol, thymol, rosmarinic acid and caffeic acid, which exhibit potent antioxidant and antimicrobial activities [18,19,20].

Oregano (*Origanum vulgare* L.) is widely used as a culinary herb to enhance the flavor of diverse foods across the globe [21]. Scientific investigations highlight phenolic compounds and terpenes as its principal bioactive constituents. For instance, rosmarinic acid, 4-(3,4-dihydroxybenzoyloxymethyl)phenyl-β-d-glucopyranoside, catechin and luteolin-7-O-rutinoside have been identified in oregano extracts [22].

Rosemary (*Rosmarinus officinalis* L.) is another member of this botanical family, distinguished by a wide spectrum of nutritionally valuable and bioactive compounds. Its primary constituents include flavonoids, phenolic acids, terpenes and essential oils. Rosemary displays remarkable antioxidant potential, largely attributed to its wealth of polyphenols, flavonoids, terpenoids and lignans. Its extracts additionally provide phenolic acids (rosmarinic, caffeic, ferulic and chlorogenic acids), diterpenes (carnosic acid, carnosol, rosmanol, rosmaridiphenol), triterpenes, flavonoids, phytosterols and a wide range of natural antioxidants [23,24,25]. Rosemary is also a source of tannins, saponins, and vitamins A, C and B6, as well as minerals such as calcium, magnesium, iron and potassium [26]. Its antimicrobial efficacy encompasses both Gram-positive and Gram-negative bacteria, though stronger activity is generally observed against Gram-positive strains. The mechanism involves disruption of cell wall and cytoplasmic membrane integrity and inhibition of nucleic acid and protein synthesis. As a result, rosemary extracts and essential oils can significantly reduce the prevalence of major foodborne pathogens, including *Listeria monocytogenes*, *Salmonella typhimurium*, *Escherichia coli* O157:H7, *Shigella dysenteriae, Bacillus cereus* and *Staphylococcus aureus* [27,28]. Similarly, oregano extracts rich in phenolic compounds and terpenes have been linked to enhanced shelf-life of meat products. For example, in lamb burgers, the inclusion of oregano extract reduced oxidative degradation of lipid and protein fractions throughout storage at −18 °C for 120 days, while maintaining a higher proportion of red color (a*) relative to controls [22]. In another study, the combined use of oregano essential oil with green tea extract (1%) improved the sensory stability of sliced cooked ham stored for 21 days at 2 °C. Samples treated with this combination exhibited more favorable color retention, reduced surface discoloration and better odor attributes throughout storage.

The aim of this study was to determine the effect of rosemary and oregano extract on selected quality parameters of pork pâtés during two weeks of cold storage. The chemical composition, physicochemical parameters, including pH, lipid oxidation rate (TBARS), oxidation-reduction potential, color and texture parameters, and sensory characteristics of these products were analyzed. The innovative nature of this study lies in the simultaneous use of rosemary and oregano extracts—plants rich in phenolic compounds with strong antioxidant and antimicrobial potential—in the formulation of refrigerated pork pâtés. To date, these extracts have been more extensively studied in products with longer shelf lives (e.g., fermented sausages, cured meats) or in the form of essential oils, but little attention has been paid to their use in pâtés, which, due to their fragmented structure and high fat content, are particularly susceptible to oxidative processes and quality loss. This research also fits into the innovative directions of development of food technology, corresponding to the “clean label” trend and the growing consumer interest in products with a higher degree of naturalness and health-promoting properties.

## 2. Results and Discussion

### 2.1. Chemical Composition

The data in Table 1 indicate that the content of individual chemical components did not change significantly (*p* < 0.05) after the addition of the extract. The fat content in the tested pâtés ranged from 19.3% to 21.4%, and the protein content from 16.5% to 17.2%. The water content remained constant at 60.0% to 61.2% throughout the cold storage period. The obtained results show that the use of oregano and rosemary extracts in various concentrations did not affect the basic chemical composition of the pâtés. These results are consistent with previous studies conducted on liver pâtés, where the use of plant extracts and essential oils as natural antioxidants did not cause changes in the chemical composition of the meat products [29,30,31]. In the study conducted by Glišić et al. [32], no significant differences were found in the content of water, protein and fat in pork pâtés with the addition of extracts from sunflower and corn crop residues.

### 2.2. Physicochemical Properties

The effect of adding rosemary and oregano extracts on pH, TBARS value, and oxidation-reduction potential in pork pâtés during cold storage is presented in Table 2.

Acidity analysis of the experimental samples did not reveal any statistically significant differences (*p* < 0.05) in pH values throughout the cold storage period. The differences in mean values of this parameter between the analyzed groups were small and ranged from 6.29 to 6.42, which is considered typical for this type of product [33]. The observed differences in pH values may be primarily due to the presence of phenolic compounds in rosemary and oregano extracts, which exhibit buffering and antimicrobial properties. These compounds may reduce acidity by inhibiting lactic acid bacteria and slowing oxidative processes, resulting in a slightly higher and more stable pH during storage. A similar trend was demonstrated in their study by Lorenzo et al. [34]. The authors observed that the pH values of the tested “Chorizo” sausages did not change with the addition of antioxidants, although the highest pH values occurred in the control group, followed by the BHT group and the group with added chestnut extract. Fernandes et al. [7] also did not observe any effect of oregano extract on the pH of lamb burgers. A similar trend was demonstrated in a study by Vergard et al. [35], who investigated the effect of adding different forms of oregano on the quality of lamb burgers over the storage period. The authors showed that the pH value was generally stable in each analyzed batch up to day 10 of storage. Slightly different results were obtained by Glišić et al. [32], who analyzed the effect of extracts from sunflower and corn crop residues as a new ingredient on the quality properties of pork liver pâtés. The authors demonstrated that the addition of plant extracts caused a significant decrease in pH compared to control and BHT pâtés (*p* < 0.01) over the 90-day storage period. Despite these statistically significant differences, changes in this parameter were small, ranging from 6.33 to 6.52. Pateiro et al. [36] also observed a similar effect of pH changes during a 24-week storage period in pork liver pâtés with 0.1% tea, chestnut, and grape seed extracts. However, no significant changes in pH were observed in pork liver pâtés enriched with seaweed, beer residue, or chestnut leaf extracts, suggesting that the dominant bioactive compounds, mainly phenolic acids, did not affect the product’s pH [29,37].

Meat-derived products, particularly pâtés, are more susceptible to oxidative deterioration than fresh meat [38,39]. This increased vulnerability is largely associated with technological operations such as grinding and thermal processing. These treatments enhance the exposure of free fatty acids to oxygen, while the presence of heat and metalloproteins further accelerates oxidative reactions by acting as catalysts [40]. Among lipid degradation products, malondialdehyde is regarded as especially important, as it serves as one of the key biomarkers of lipid peroxidation. Its quantification is most commonly performed using the TBARS assay (thiobarbituric acid reactive substances) [33,41]. Based on the conducted studies (Table 2), a statistically significant (*p* < 0.05) effect of rosemary and oregano extract on the TBARS index was found throughout the cold storage period. However, no significant differences (*p* < 0.05) in this index were found between the control sample and the “rr” and “rs” samples on the 14th day of storage. As storage time progressed, the TBARS index values increased statistically significantly (*p* < 0.05) in almost all samples tested. The greatest changes in fat oxidation throughout the entire study period were observed for the “oo” sample. This sample had the highest TBARS value on the 14th day of storage—2.61 mg MDA/kg of product. Throughout the entire cold storage period, the lowest TBARS values were observed for the pâté with 50% rosemary extract. Two-factor analysis of variance showed that the extract type, storage time, and the interaction effect between storage time and extract type had statistically significant effects (*p* < 0.05) on the TBARS value. The antioxidant efficacy of rosemary and oregano extracts stems from the presence of phenolic compounds that interfere with lipid peroxidation at various process stages. Their primary mechanism of action is based on their ability to donate hydrogen atoms or electrons to lipid-free radicals, terminating radical chain reactions and preventing the propagation phase of lipid oxidation. Furthermore, these compounds exhibit strong metal chelating properties, reducing the prooxidant activity of transition metals such as Fe^2+^ and Cu^2+^, which catalyze the breakdown of lipid hydroperoxides into byproducts, including malondialdehyde. Naturally occurring phenolic diterpenes in rosemary, such as carnosic acid and carnosol, also stabilize lipid structures by interacting with the lipid-water interface, thereby enhancing the integrity of cell membranes and the product’s oxidative resistance. Oregano extract, rich in rosmarinic acid and flavonoids, supports antioxidant protection by effectively scavenging reactive oxygen species and inhibiting enzymatic systems involved in lipid degradation [8,42,43,44]. According to Sebranek et al. [44], rosemary extract used in the production of boiled sausages limits lipid oxidation and protects the product’s color as effectively as BHA and BHT. Studies conducted by Pietrzak and Myron [45] on hamburgers found that adding rosemary extract slowed fat oxidation reactions. Erkan et al. [43] demonstrated that rosemary extract is particularly rich in phenolic compounds, which accounts for its pronounced antioxidant capacity. Among these, several phenolic diterpenes—such as carnosic acid, carnosol, rosmanol, rosmariquinone, and rosmaridiphenol—contribute to this effect. Their mode of action is associated with the interruption of free radical chain reactions through the donation of hydrogen atoms, thereby enhancing the antioxidant potential of rosemary-derived extracts. Furthermore, a study by Fernandes et al. [7] demonstrated that the addition of oregano extract exerted an antioxidant effect by reducing lipid oxidation. A similar trend was demonstrated in a study by Deng et al. [46], who investigated the effect of green tea and its polyphenols on the formation of heterocyclic aromatic amines, antioxidant capacity, and quality characteristics of roasted pork cutlets. The authors demonstrated that the addition of green tea extract (0.05%, 0.25%, and 0.5%) significantly reduced the TBARS value (*p* < 0.05), with inhibition rates in the ranges 91.50–95.00%, 93.72–95.20%, and 93.65–97.32%, respectively. Furthermore, the reduction effect increased with increasing additive levels. Camo et al. [47] investigated the influence of oregano extract on the antioxidant properties of fresh beef steaks stored under an active packaging system. The incorporation of 1% oregano extract extended the shelf life from 14 to 23 days, whereas a 4% concentration led to an undesirable oregano odor within the first day. Optimal packaging performance was achieved with oregano extract concentrations of 1% and 2%. The study conducted by Tang et al. [48] also demonstrated a beneficial effect of the addition of tea catechins (TC) on the TBARS index in ground beef packaged under aerobic conditions and in MAP.

One of the primary factors influencing meat coloration is the redox potential, which governs the oxidation state of the iron atom located within the porphyrin ring of the myoglobin molecule [49,50]. The oxidation-reduction potential (ORP) values of the tested pork pâtés differed significantly between samples and during cold storage (*p* < 0.05). After 1 day of storage, statistically significant differences were found between the control sample and those with the extract added, and between the samples with rosemary extract and those with oregano extract added. After 7 days, ORP values in all samples were similar (316.25–324.92 mV), and the differences were statistically insignificant, indicating that this parameter leveled off throughout storage. After 14 days, a further difference was observed—ORP in the control sample reached the highest value (382.42 mV), while the lowest value was in the “os” sample (316.03 mV). The “rr” sample had the lowest redox potential after 1 day of storage (300.43 mV). This value was significantly lower (*p* < 0.05) than the control sample and the “oo” and “os” samples. Furthermore, the “rr” sample was characterized by the smallest changes in redox potential throughout the storage period. Extract type, storage time, and the interaction effect between storage time and extract type had a statistically significant effect on the redox potential value. The obtained results suggest the possibility of synergistic or antagonistic interactions between rosemary and oregano extracts. Due to their distinct phytochemical profiles—rosemary being rich in phenolic diterpenes such as carnosic acid and carnosol, and oregano containing monoterpenes and phenolic acids like thymol and rosmarinic acid—these compounds may act complementarily, strengthening the antioxidant protection of the product. A balanced combination of both extracts could therefore enhance oxidative stability and further reduce lipid oxidation. However, excessive concentrations, particularly of oregano, may result in antagonistic effects manifested by an overly intense herbal aroma or diminished antioxidant efficiency. Future studies should verify these potential interactions and establish the most effective extract ratio for meat product applications.

### 2.3. Color Parameters

Table 3 presents the results of the analysis of the effect of rosemary and oregano extract on color parameters L* (brightness), a* (redness), b* (yellowness), browning index (BI), and absolute color difference. The values of the L* parameter, reflecting the color lightness of the tested pâtés, showed significant variation depending on storage time and the type of extract added. After 1 day of cold storage, the L* values of the analyzed samples ranged from 56.77 to 59.05. The lowest color lightness was found in the “os” sample (56.77), which differed significantly (*p* < 0.05) from the other groups, while the highest was found in the control sample. With increasing storage time, an increase in color lightness (L*) was observed for all analyzed samples. The highest color lightness values were observed for the “k”, “rr”, “rs” and “oo” samples. These samples were significantly lighter (*p* < 0.05) than the “os” samples. The obtained results suggest that the dynamics of brightness changes may be related to oxidative processes and degradation of natural dyes, which is consistent with previous literature reports on the storage of products with different colors [38,39]. Statistical analysis of the color test results showed no significant effect of rosemary and oregano extract addition or cold storage time on changes in the a* parameter value (determining the proportion of red color) of the tested pork pâtés. Lower a* parameter values were observed in the pâtés with rosemary and oregano extract compared to the control sample throughout the entire study period. This can be assumed to be due to the green color of the extract. Statistical analysis of the color test results also revealed no significant effect of rosemary and oregano extract and cold storage time on changes in the b* parameter (determining the share of yellow color) of the tested pâtés. However, the interaction effect between storage time and extract type was found to have a statistically significant effect on changes in the b* value. The most pronounced differences were observed in the “rs” and “oo” samples, where cold storage time led to a significant decrease in this parameter. This phenomenon may be related to the degradation of the color compounds responsible for the yellow color. Two-factor analysis of variance showed that both extract type, storage time, and the interaction effect between storage time and extract type had a statistically significant effect (*p* < 0.05) on the browning index value. The general trend indicates a decrease in the degree of browning with storage time, which may be related to the degradation processes of the dyes responsible for darker shades of color. Contrasting observations were reported by Pateiro et al. [51], who demonstrated that the incorporation of tea extract and grape seed extract into pork liver pâtés led to variable alterations in product color. Similarly, the study of Yu et al. [52] on the antioxidant activity of an aqueous rosemary extract confirmed that increasing its concentration resulted in progressive darkening of cooked poultry products. In line with this, Fernández-López et al. [53] observed that supplementing beef meatballs with extracts of rosemary, garlic, orange, and lemon slightly reduced product lightness. A comparable trend was noted in poultry burgers enriched with Scutellaria baicalensis (Baikal skullcap), where higher supplementation levels corresponded with a decline in brightness [54]. Findings comparable to those obtained in the present study were reported by Pateiro et al. [36], who noted an increase in lightness (L*) and a reduction in redness (a*) in pork liver pâtés formulated with green tea and chestnut extracts, whereas no significant changes were observed in the control samples during cold storage. These results are also consistent with earlier observations by Estévez and Cava [38] and Estévez et al. [39], who documented deterioration in color attributes of pâtés under chilled conditions. In their studies, a rise in L* values accompanied by a decline in both a* and b* coordinates was recorded during cold storage.

### 2.4. Texture Parameters

Food texture encompasses a set of physical characteristics inherently linked to the structural organization of a product at the molecular, microscopic, and macroscopic levels. These attributes are generally classified into mechanical and rheological properties. Texture profile analysis parameters reflect not only elasticity and surface-related features, but also the internal structural traits of the product, as they capture a range of physical phenomena occurring during measurements that involve substantial material deformation [33,55]. The results regarding the texture parameters of pork pâtés with and without the addition of oregano and rosemary extract are presented in Table 4. Our own research on the analysis of the texture profile of pâtés showed that replacing water with rosemary and oregano extract, rich in substances with antioxidant properties, causes significant differences in the values obtained from the measurement of such parameters as: hardness in cycles 1 and 2, adhesiveness, springiness and chewiness. No statistically significant differences were found for the remaining texture parameters, i.e., resilience and cohesiveness. It was found that cold storage time increased cycle 1 and cycle 2 hardness, adhesiveness, springiness, and chewiness. Furthermore, the addition of oregano and rosemary extract significantly increased the springiness of the tested pâtés on the 7th day of cold storage, after which this characteristic decreased on the 14th day of storage in all analyzed samples. Two-factor analysis of variance revealed an interaction effect of cold storage time and extract addition for texture parameters such as cycle 1 and cycle 2 hardness, springiness, and chewiness. Salejda et al. [56] reported that the incorporation of green tea extract influenced the texture of model meat batters, showing a significant reduction in hardness as well as decreases in springiness and cohesiveness. A comparable trend concerning hardness was observed by Kliks et al. [57], who analyzed the effect of green tea extract on selected properties of finely comminuted sausages. Their findings indicated that formulations enriched with the extract exhibited lower hardness compared to the control group, while a 5% supplementation level additionally reduced springiness. In contrast, Deng et al. [46] demonstrated that the addition of green tea to baked pork patties had no significant effect on textural parameters, including hardness, springiness, cohesiveness, or chewiness.

### 2.5. Sensory Properties

Lipid and protein oxidation represents the second most critical cause of meat spoilage, following microbial deterioration, and exerts a detrimental impact on the sensory quality of meat products [58]. The outcomes of the sensory evaluation of pork pâtés, depending on the type of extract applied and the duration of cold storage, are presented in Table 5. For most of the assessed sensory attributes, the pork pâté with various extracts obtained higher scores throughout the storage period compared to the control sample. The study demonstrated a statistically significant (*p* < 0.05) effect of oregano and rosemary extract on the variability of sensory properties such as odor, taste, and color. In the case of appearance and consistency, no significant differences (*p* < 0.05) were found between the analyzed samples, and the obtained scores remained stable throughout the storage period. Similar results were obtained by Kliks et al. [57], who also found no significant effect of green tea extracts at various concentrations on the overall appearance of finely ground sausage. According to Tolik et al. [59], the assessment of consistency is primarily influenced by the water and fat content of meat products. Fat primarily shapes the consistency of pâtés, and low amounts of it cause them to become drier and stiffer, and increases gumminess.

Sensory evaluation of the tested pâtés revealed significant differences in odor between the samples (*p* < 0.05). On the first day of storage, the “rs” and “os” samples received the highest scores (4.58 and 4.52 points, respectively), differing significantly from the other samples. A similar trend was observed after 7 days of cold storage, with the “rs” and “os” samples achieving the highest scores (4.58), significantly exceeding the values obtained for the other samples. After 14 days of storage, differences between the samples were still evident—the highest scores were recorded for the “rs” and “os” samples, while the other variants achieved lower scores. The results indicate that the modifications applied to the “rs” and “os” samples had a beneficial effect on odor perception, and this effect persisted throughout the storage period. Taste evaluation also showed significant differences between the tested samples (*p* < 0.05). On the first day of storage, the highest scores were achieved by samples with added rosemary and oregano extract (4.50–4.67), which were rated significantly better compared to the control sample (3.92). A similar trend continued after 7 days of cold storage, with the “oo” sample receiving the highest score (4.75). After 14 days of storage, a decrease in scores was observed for the control sample, while the remaining samples maintained relatively high acceptance within the 4.17–4.50 point range. The results indicate that the addition of rosemary and oregano extracts positively influenced taste perception, and this effect persisted throughout the storage period. Similar trends were observed when assessing the color of the tested pâtés. The color of most samples was shown to be relatively stable throughout the study period, but in the “oo” sample, a significant decline in this parameter was observed after 14 days of storage. Furthermore, the addition of oregano and rosemary extracts has been shown to have a beneficial effect on selected sensory properties of pork pâtés during cold storage. The most noticeable changes were in odor and taste, which may be attributed to the presence of phenolic compounds such as rosmarinic acid, carnosol, carnosinate, and thymol, which limit the formation of undesirable aromas resulting from oxidative processes. Similar results were obtained in the study by Al-Hijazeen and Al-Rawashdeh [60]. The authors demonstrated that the addition of rosemary significantly improved the odor rating of the tested poultry pâtés compared to the control sample and samples with added artificial antioxidants. Furthermore, Fernandes et al. [7] demonstrated that the addition of oregano extract improved the sensory stability of sheep burgers packaged in MAP for up to 15 days of cold storage. Danilović et al. [61] emphasized that oils and extracts can alter the aroma and flavor, and therefore should be used at the lowest possible concentration. However, the concentration must still be sufficient to achieve the desired effects, such as antioxidant or antimicrobial activity, extension of product shelf life, and other beneficial properties. Numerous studies have demonstrated that natural antioxidants can influence the color and sensory attributes of meat products in both beneficial and adverse ways. The activity and effectiveness of plant extracts are further affected by factors such as the extraction technique, processing conditions applied to the samples, and treatments carried out after packaging [62].

## 3. Materials and Methods

### 3.1. Preparation of Extracts

Dried oregano (*Origanum vulgare*) and rosemary (*Rosmarinus officinalis*) leaves were purchased from an organic food store. The raw materials were certified organic. The material was vacuum-packed and stored at room temperature in a dry, dark place until the extracts were prepared. To obtain the oregano and rosemary extract, the methodology described by Michiels et al. [63] was used, with some modifications. A solvent mixture (acetone/water/glacial acetic acid, 70:28:2% *v*/*v*) was used for extraction at a ratio of 1:20 (g of plant material per ml of solvent mixture). The dried leaves were homogenized in this solvent for 2 min using a T25 digital Ultra Turrax homogenizer (IKA, Königswinter, Germany). The mixture was shaken for 1 h at 50 rpm and then centrifuged (MPW-352R, Warsaw, Poland) for 15 min at 4000 rpm. The supernatant was passed through filter paper (Whatman, Maidstone, UK). After filtration, the supernatant was evaporated under reduced pressure at 40 °C using a rotary evaporator (Heidolph, Schwabach, Germany) to completely remove the solvent. The extract was then stored refrigerated in airtight containers until analysis. Before testing, the extract was diluted 1:4 with water. The oregano and rosemary extraction process was performed three times.

### 3.2. Preparation of Pork Pâtés

The research material consisted of baked pork pâtés made from a meat-fat filling, with or without the addition of extracts. Six batches of pâtés were produced, and three samples were taken from each batch. The pâtés consisted of meat and fat ingredients: pork liver (30%), pork jowl (30%), pork rinds (20%), water or extract (10%), and non-meat additives: wheat bread, salt, onion, pepper, and eggs (10%) (Table 6). The meat and fat ingredients were purchased from the company store of a meat processing plant in southeastern Poland. The ingredients were trimmed of visible fat and connective tissue and pre-treated. The pork liver was scalded for 10 min at 75 °C, while the pork jowl and rinds were cooked until tender. All ingredients were then ground in a laboratory mill using a Royal Catering RCMM-2000 grinder (Royal Catering, Butzbach, Germany), equipped with a 4 mm perforated plate to achieve the desired particle size, and then homogenized (8000 rpm, 5 min) on a Servotech device (ZB-80, Hamburg, Germany). The final temperature of the batters was below 15 °C in all cases. Five different experimental variants were prepared: “k”—control sample (no extract added), “rr”—sample with 50% rosemary extract, “rs”—sample with 100% rosemary extract, “oo”—sample with 50% oregano extract, and “os”—sample with 100% oregano extract. The resulting mixture was then filled into aluminum molds measuring 207 mm × 84 mm × 52 mm. The pâtés were baked at 180 °C until a temperature of 75 °C was reached inside the meat bar. After baking, the cooled pâtés were vacuum-packed using multilayer PA/PE foil using an Inauen VC999 vacuum packaging machine (Inauen, Herisau, Switzerland). Throughout the entire study period, the pâtés were stored cold at ±4 °C. The analyses specified in the experiment were conducted after 1, 7, and 14 days of cold storage. After each period of cold storage, all variants were subjected to chemical composition analysis, pH, oxidation-reduction potential, TBARS, color determination, texture parameters and sensory properties.

### 3.3. Research Methods

#### 3.3.1. Chemical Composition Analysis

Prior to the analysis of chemical composition, the samples were homogenized using a laboratory meat grinder (Royal Catering, RCMM-2000, Butzbach, Germany) equipped with a 4 mm perforated plate. The determination of water content was performed in accordance with the PN-ISO 1442:2000 standard [64]. Protein content was analyzed following the guidelines specified in PN-75/A-04018:2002 [65], while fat content was measured according to the PN-ISO 1444:2000 standard [66].

#### 3.3.2. Physicochemical Properties

pH measurement was carried out using a CPC-411 pH meter (ELMETRON, Zabrze, Poland) equipped with an OSH 12-01 electrode and an automatic temperature compensation system, ensuring a measurement accuracy of ±0.01. The device was calibrated at 20 °C using standard buffer solutions with pH values of 4.00 and 7.00. pH was measured immediately following the color evaluation.

The evaluation of the TBARS index involved quantifying a group of compounds capable of forming colored complexes, primarily with 2-thiobarbituric acid, including malondialdehyde. This method is based on the formation of intensely colored complexes generated by aldehydes present in lipids when reacted with a 2-thiobarbituric acid solution under elevated temperature conditions. The resulting coloration from the reaction mixture, consisting of the meat product sample and 2-thiobarbituric acid, was measured spectrophotometrically at 532 nm using a Spekol 2000 spectrophotometer (Analytik Jena AG, Kundendienst, Jena, Germany). A control sample, following the procedure described by Pikul, Leszczyński, and Kumerow [67] and Duma-Kocan et al. [49], was used as the reference.

The oxidation-reduction potential (EH, mV) was measured using an ERPt-13 combination electrode paired with a waterproof pH/conductivity meter, namely the ELMETRON CPC-505 device (Zabrze, Poland).

#### 3.3.3. Instrumental Evaluation of Color

Sample surfaces were sliced and allowed to bloom for 30 min prior to color assessment. Instrumental color measurements were conducted on meat cross-sections using a HunterLab UltraScan PRO electronic colorimeter (HunterLab, Reston, VA, USA) with illuminant D65 and a 20 mm aperture. Calibration of the device was performed using a white standard with values of L* = 99.18, a* = 0.07, and b* = 0.05. After the blooming period, color evaluation was carried out as described above. For each sample, three replicate measurements were taken at different locations on the same surface, and the mean value was used for further analysis. The browning index (BI) was calculated following the method reported by Pérez-López et al. [68]. Furthermore, the total color difference (∆E) between control samples and type of extract was computed based on the obtained color parameters, applying the procedure described by Cserhalmi et al. [69].

#### 3.3.4. Texture Profile Analysis (TPA)

The assessment of texture parameters was performed following established protocols described in the literature, particularly those by Jaico et al. [70] and Ma and Ledward [71]. Instrumental texture analysis was performed using a CT3-25 texture analyzer (AMETEK, Brookfield, WI, USA) on meat samples, which were prepared as cubes measuring 20 mm on each side and equipped with a cylindrical probe. A two-cycle compression test was applied, compressing the samples to 50% of their initial height. The probe operated at a speed of 2 mm/s, with a 2 s pause between compressions. All measurements were conducted at a sample temperature of 20 ± 1 °C to ensure consistency and comparability of texture data, as mechanical properties of meat are highly temperature-dependent and can vary significantly with thermal fluctuations. Texture parameters were determined using Texture Pro CT software V.1.9. Build 39 (AMETEK, Brookfield, WI, USA). For each measurement, the mean value was calculated from six consecutive repetitions to obtain the final texture parameter.

#### 3.3.5. Sensory Evaluation

All samples were placed in sealed plastic containers marked with unique digital codes. Sensory evaluation was performed at room temperature in individual white light booths during the day. Samples were selected for sensory evaluation in random order. Sensory evaluations were performed by a permanent laboratory team consisting of ten panelists, each experienced in evaluating meat and meat products. The team followed the principles specified in ISO 8586-2 [72], focusing on sensitivity and sensory parameters. The sensory evaluation was conducted in accordance with ISO 13299:2016 [73]. The evaluation panel consisted of individuals aged 24 to 45 years. The evaluators were proficient in evaluating the sensory attributes of meat and meat products. A 5-point partial quality scale was used for the sensory evaluation, and the evaluated attributes are described in detail in Table 7. Before each sample was tested, the evaluators took a 30 s break and rinsed their mouths with mineral water.

### 3.4. Statistical Analysis

All observations obtained in the experiment (2 extract × 6 batches × 3 storage periods) were included in the statistical analysis. The distribution of the data was assessed for normality using the Kolmogorov–Smirnov test, while the homogeneity of variances was evaluated with the Brown–Forsythe test. Selected physicochemical parameters, along with texture and sensory attributes of the pork pate, were analyzed using two-way analysis of variance (ANOVA), implemented through the General Linear Model (GLM) procedure in Statistica (STATISTICA, version 13.3; StatSoft, Krakow, Poland). In this model, extract type and storage duration were considered fixed effects, whereas batch was treated as a random effect. For sensory data, the model also incorporated panelist as a factor and included interactions between the main effects. Batch was used as the error term to evaluate the significance of the fixed effects and their interaction. Post hoc comparisons were conducted using Tukey’s test, with statistical significance set at *p* < 0.05. Mean values and standard errors of the mean (SEM) were reported for all variables (as presented in the tables). All statistical analyses were performed using the GLM procedure in Statistica (STATISTICA, version 13.3; StatSoft, Krakow, Poland).

## 4. Conclusions

The study showed that the addition of rosemary and oregano extracts to pork pâtés did not alter the basic chemical composition or pH, but significantly (*p* < 0.05) affected the oxidative stability, color, texture parameters, and sensory evaluation of the tested samples. TBARS values increased during storage of these products, with the lowest rate of lipid oxidation observed in samples with rosemary extract. Oregano and rosemary extracts limited the increase in the oxidation-reduction potential of the pâtés compared to the control sample. Changes in the texture parameters of the pâtés were observed during storage, with the additives significantly limiting unfavorable changes, particularly in hardness and chewiness. Sensory analysis revealed a positive effect of both extracts, particularly in terms of odor and taste perception, which were rated significantly higher than the control sample. The use of these extracts may enable partial or complete replacement of synthetic antioxidants and naturally extend the shelf life of meat products. Further research is needed to confirm these findings in industrial settings and to evaluate other natural extracts or their combinations for longer shelf life.

## Figures and Tables

**Table 1:** The chemical composition of pork pâtés depends on the type of extract and cold storage time.

Parameters	Time [Days]	k	Extract				SEM	ANOVA	*p*-Value
			rr	rs	oo	os			
Fat (%)	1	21.42	21.20	20.57	20.48	20.15	0.60	-	-
	7	20.60	20.32	21.13	19.93	20.57	0.20		
	14	20.48	19.33	20.42	20.28	20.37	0.31		
	SEM	0.26	0.28	0.36	0.80	0.40			
Water (%)	1	60.02	60.18	60.68	60.73	61.00	0.48	-	-
	7	60.73	60.43	60.27	61.22	60.32	0.15		
	14	60.78	60.55	60.65	60.92	60.47	0.32		
	SEM	0.22	0.25	0.30	0.19	0.31			
Protein (%)	1	16.48	16.52	16.65	16.70	17.03	0.15	-	-
	7	16.65	16.72	16.55	16.83	17.13	0.07		
	14	16.68	16.88	16.71	16.75	17.18	0.09		
	SEM	0.07	0.10	0.10	0.08	0.11			

Explanatory notes: “k”—control sample (no extract added), “rr”—with 50% rosemary extract added, “rs”—with 100% rosemary extract added, “oo”—with 50% oregano extract added, “os”—with 100% oregano extract added; SEM—standard error of mean; ANOVA—two-factor analysis of variance between the refrigerated storage time (T) and extract used (Z), *p* < 0.05. There were no statistically significant differences between the groups.

**Table 2:** Physicochemical properties of pork pâtés depend on the type of extract and cold storage time.

Parameters	Time [Days]	k	Extract				SEM	ANOVA	*p*-Value
			rr	rs	oo	os			
pH	1	6.34 ^a^	6.36 ^a^	6.40 ^a^	6.29 ^a^	6.37 ^a^	0.02	-	-
	7	6.33 ^a^	6.33 ^a^	6.35 ^a^	6.35 ^a^	6.35 ^a^	0.03		
	14	6.38 ^a^	6.42 ^a^	6.37 ^a^	6.33 ^a^	6.43 ^a^	0.04		
	SEM	0.02	0.02	0.02	0.01	0.06			
TBARS index (mg MDA/kg)	1	1.58 ^ax^	1.34 ^bx^	1.33 ^bx^	1.87 ^cx^	1.80 ^cx^	0.02	Z T ZxT	<0.0001 <0.0001 <0.0001
	7	1.80 ^ay^	1.73 ^ay^	1.99 ^by^	1.87 ^acx^	2.07 ^bcy^	0.06		
	14	2.12 ^az^	1.90 ^az^	2.17 ^az^	2.61 ^by^	2.32 ^cz^	0.06		
	SEM	0.03	0.04	0.04	0.06	0.07			
Oxidation-reduction potential (mV)	1	314.22 ^ax^	300.43 ^bx^	300.55 ^bx^	384.12 ^cx^	376.10 ^bcx^	1.13	Z T ZxT	<0.0001 <0.0001 <0.0001
	7	320.20 ^ay^	318.78 ^ay^	324.92 ^ay^	319.27 ^ay^	316.25 ^ay^	1.18		
	14	382.42 ^az^	352.98 ^bz^	353.92 ^bz^	326.12 ^bcy^	316.03 ^bcy^	1.42		
	SEM	1.44	1.43	2.53	0.71	0.62			

Explanatory notes: “k”—control sample (no extract added), “rr”—with 50% rosemary extract added, “rs”—with 100% rosemary extract added, “oo”—with 50% oregano extract added, and “os”—with 100% oregano extract added; ^a,b,c^—differences in the same rows are statistically significant at the level *p* < 0.05, and no letters or the same letters indicate no statistically significant differences; ^x,y,z^—differences in the same columns are statistically significant at the level *p* < 0.05, and no letters or the same letters indicate no statistically significant differences; SEM—standard error of mean; ANOVA: two-factor analysis of variance between the refrigerated storage time (T) and extract used (Z), *p* < 0.05.

**Table 3:** Color parameters of pork pâtés depending on the extract and cold storage time.

Parameters	Time [Days]	k	Extract				SEM	ANOVA	*p*-Value
			rr	rs	oo	os			
L*	1	59.05 ^a^	58.42 ^ax^	57.96 ^abx^	58.20 ^a^	56.77 ^b^	0.98	ZxT	0.4755
	7	59.71 ^a^	59.13 ^ax^	59.50 ^ax^	57.38 ^b^	57.16 ^b^	1.48		
	14	61.32 ^a^	60.92 ^ay^	61.16 ^ay^	60.15 ^a^	58.55 ^b^	0.78		
	SEM	0.85	0.91	0.87	1.42	1.30			
a*	1	12.97 ^a^	12.80 ^a^	12.64 ^a^	12.88 ^a^	12.52 ^a^	0.51	-	-
	7	12.57 ^a^	12.31 ^a^	12.07 ^a^	12.61 ^a^	12.61 ^a^	0.49		
	14	12.97 ^a^	12.27 ^a^	12.69 ^a^	12.57 ^a^	12.95 ^a^	0.41		
	SEM	0.42	0.40	0.43	0.62	0.47			
b*	1	12.46 ^a^	13.11 ^a^	13.29 ^ax^	13.30 ^ax^	12.57 ^a^	0.51	ZxT	<0.0001
	7	12.77 ^a^	12.90 ^a^	12.61 ^ax^	11.98 ^ay^	11.92 ^a^	0.45		
	14	12.08 ^a^	12.37 ^a^	11.75 ^ay^	11.81 ^ay^	11.69 ^a^	0.38		
	SEM	0.42	0.49	0.51	0.48	0.31			
BI	1	39.22 ^ax^	40.91 ^ax^	41.48 ^bx^	41.61 ^bx^	41.67 ^bx^	1.24	Z T ZxT	0.0528 <0.0001 0.0049
	7	38.94 ^ax^	39.36 ^ax^	38.16 ^ay^	38.99 ^ay^	38.97 ^ax^	1.05		
	14	36.88 ^ay^	36.94 ^ay^	35.99 ^ay^	36.61 ^ay^	37.88 ^ay^	0.72		
	SEM	0.69	1.07	0.95	1.10	0.95			
∆E	1		0.92	1.41	1.20	2.33			
	7		0.65	0.57	2.46	2.69			
	14		0.86	0.46	1.27	2.80			

Explanatory notes: “k”—control sample (no extract added), “rr”—with 50% rosemary extract added, “rs”—with 100% rosemary extract added, “oo”—with 50% oregano extract added, and “os”—with 100% oregano extract added; ^a,b^—differences in the same rows are statistically significant at the level *p* < 0.05, and no letters or the same letters indicate no statistically significant differences; ^x,y^—differences in the same columns are statistically significant at the level *p* < 0.05, and no letters or the same letters indicate no statistically significant differences; SEM—standard error of mean; ANOVA: two-factor analysis of variance between the refrigerated storage time (T) and extract used (Z), *p* < 0.05.

**Table 4:** Texture parameters depend on the type of extract and cold storage time.

Parameters	Time [Days]	k	Extract				SEM	ANOVA	*p*-Value
			rr	rs	oo	os			
Hardness cycle 1 [N]	1	11.10 ^ax^	11.24 ^ax^	9.65 ^ax^	10.36 ^ax^	12.69 ^a^	1.64	T ZxT	<0.0001 <0.0001
	7	16.24 ^ay^	17.69 ^ay^	19.88 ^ay^	13.54 ^bx^	12.02 ^b^	1.74		
	14	18.54 ^ay^	17.25 ^ay^	15.95 ^by^	14.32 ^by^	15.77 ^b^	1.73		
	SEM	1.73	1.53	1.20	1.65	1.98			
Hardness cycle 2 [N]	1	9.31 ^ax^	9.38 ^ax^	7.91 ^ax^	8.56 ^ax^	10.57 ^ax^	1.52	T ZxT	<0.0001 <0.0001
	7	13.39 ^ay^	14.69 ^ay^	16.26 ^ay^	11.28 ^by^	10.12 ^bx^	1.50		
	14	15.15 ^ay^	13.92 ^ay^	13.17 ^ay^	11.80 ^ay^	13.28 ^ay^	1.53		
	SEM	1.53	1.36	0.90	1.41 ^a^	1.66 ^a^			
Resilience	1	0.13 ^a^	0.13 ^a^	0.14 ^a^	0.16 ^a^	0.13 ^a^	0.02	-	-
	7	0.16 ^a^	0.15 ^a^	0.13 ^a^	0.16 ^a^	0.15 ^a^	0.02		
	14	0.08 ^a^	0.08 ^a^	0.09 ^a^	0.08 ^a^	0.18 ^a^	0.05		
	SEM	0.02	0.02	0.01	0.02	0.08			
Adhesiveness [mJ]	1	0.27 ^ax^	0.42 ^ax^	0.40 ^ax^	0.73 ^ax^	0.48 ^ax^	0.33	T	<0.0001
	7	2.28 ^ay^	2.73 ^ay^	2.53 ^ay^	2.25 ^ay^	2.97 ^ay^	0.52		
	14	3.12 ^ay^	3.22 ^ay^	3.03 ^ay^	3.33 ^ay^	3.50 ^ay^	0.85		
	SEM	0.65	0.56	0.69	0.51	0.16			
Cohesiveness	1	0.31 ^a^	0.30 ^a^	0.31 ^a^	0.34 ^a^	0.33 ^a^	0.03	-	-
	7	0.37 ^a^	0.33 ^a^	0.33 ^a^	0.33 ^a^	0.32 ^a^	0.04		
	14	0.22 ^a^	0.25 ^a^	0.25 ^a^	0.25 ^a^	0.24 ^a^	0.04		
	SEM	0.03	0.03	0.03	0.05	0.04			
Springiness [mm]	1	4.42 ^ax^	4.44 ^a^	4.57 ^a^	4.63 ^a^	4.73 ^a^	0.40	ZxT	0.0464
	7	5.60 ^ay^	5.04 ^b^	4.70 ^b^	4.82 ^b^	4.63 ^b^	0.39		
	14	3.60 ^ax^	3.84 ^a^	3.81 ^a^	3.53 ^a^	3.58 ^a^	0.52		
	SEM	0.34	0.48	0.33	0.39	0.57			
Chewiness [mJ]	1	5.13 ^ax^	15.28 ^bx^	13.45 ^bx^	16.27 ^b^	18.42 ^b^	2.64	T ZxT	<0.0001 <0.0001
	7	34.02 ^ay^	29.45 ^ay^	27.50 ^aby^	21.30 ^c^	17.70 ^c^	5.03		
	14	14.95 ^ax^	16.47 ^ax^	15.45 ^ax^	11.12 ^a^	17.85 ^a^	3.25		
	SEM	4.95	4.33	3.39	2.40	3.16			

Explanatory notes: “k”—control sample (no extract added), “rr”—with 50% rosemary extract added, “rs”—with 100% rosemary extract added, “oo”—with 50% oregano extract added, and “os”—with 100% oregano extract added; ^a,b,c^—differences in the same rows are statistically significant at the level *p* < 0.05, and no letters or the same letters indicate no statistically significant differences; ^x,y^—differences in the same columns are statistically significant at the level *p* < 0.05, and no letters or the same letters indicate no statistically significant differences; SEM—standard error of mean; ANOVA: two-factor analysis of variance between the refrigerated storage time (T) and extract used (Z), *p* < 0.05.

**Table 5:** Results of sensory evaluation of pork pâtés depending on the type of the extract and cold storage time [points].

Parameters	Time [Days]	k	Extract				SEM	ANOVA	*p*-Value
			rr	rs	oo	os			
Odor	1	4.07 ^a^	4.42 ^ax^	4.58 ^b^	4.25 ^a^	4.52 ^b^	0.22	Z ZxT	*p* < 0.0001 0.0451
	7	4.08 ^a^	4.17 ^ax^	4.58 ^b^	4.17 ^a^	4.58 ^b^	0.26		
	14	4.00 ^a^	4.00 ^ay^	4.58 ^b^	4.33 ^a^	4.53 ^b^	0.13		
	SEM	0.16	0.16	0.20	0.26	0.34			
Appearance	1	4.25 ^a^	4.33 ^a^	4.33 ^a^	4.33 ^a^	4.58 ^a^	0.25	-	-
	7	4.58 ^a^	4.50 ^a^	4.58 ^a^	4.25 ^a^	4.17 ^a^	0.20		
	14	3.92 ^a^	4.17 ^a^	4.33 ^a^	4.00 ^a^	4.25 ^a^	0.20		
	SEM	0.22	0.18	0.24	0.18	0.24			
Taste	1	3.92 ^a^	4.50 ^bx^	4.50 ^b^	4.67 ^bx^	4.58 ^b^	0.27	Z ZxT	*p* < 0.0001 0.0425
	7	4.00 ^a^	4.67 ^bx^	4.50 ^b^	4.75 ^bx^	4.67 ^b^	0.26		
	14	3.50 ^a^	4.17 ^cy^	4.42 ^b^	4.00 ^cy^	4.50 ^c^	0.17		
	SEM	0.18	0.28	0.18	0.18	0.21			
Consistency	1	4.08 ^a^	4.08 ^a^	4.17 ^a^	4.25 ^a^	4.20 ^a^	0.31	-	-
	7	4.08 ^a^	4.00 ^a^	4.20 ^a^	4.17 ^a^	4.17 ^a^	0.28		
	14	4.00 ^a^	3.83 ^a^	3.92 ^a^	4.00 ^a^	4.08 ^a^	0.25		
	SEM	0.20	0.26	0.26	0.18	0.32			
Color	1	4.00 ^ax^	4.00 ^ax^	4.00 ^ax^	4.50 ^bx^	4.75 ^bx^	0.24	T ZxT	*p* < 0.0001 0.0016
	7	4.58 ^ay^	4.50 ^ay^	4.58 ^ay^	4.17 ^ay^	4.17 ^ay^	0.24		
	14	3.92 ^ax^	4.00 ^ax^	4.17 ^ax^	3.32 ^bz^	4.17 ^ay^	0.41		
	SEM	0.30	0.24	0.22	0.57	0.26			

Explanatory notes: “k”—control sample (no extract added), “rr”—with 50% rosemary extract added, “rs”—with 100% rosemary extract added, “oo”—with 50% oregano extract added, and “os”—with 100% oregano extract added; ^a,b,c^—differences in the same rows are statistically significant at the level *p* < 0.05, and no letters or the same letters indicate no statistically significant differences; ^x,y,z^—differences in the same columns are statistically significant at the level *p* < 0.05, and no letters or the same letters indicate no statistically significant differences; SEM—standard error of mean; ANOVA: two-factor analysis of variance between the refrigerated storage time (T) and extract used (Z), *p* < 0.05.

**Table 6:** Pork pâtés formulations [%].

Ingredients	“k”	“rr”	“rs”	“oo”	“os”
pork liver	30	30	30	30	30
pork jowl	30	30	30	30	30
pork rinds	20	20	20	20	20
water	10	-	-	-	-
rosemary extract 50%	-	10	-	-	-
rosemary extract 100%	-	-	10	-	-
oregano extract 50%	-	-	-	10	-
oregano extract 100%	-	-	-	-	10
non-meat additions	10	10	10	10	10

**Table 7:** The point scale for sensory evaluation of pork pâtés.

Specification	1	2	3	4	5
Odor	Unpleasant, very faint	Hardly noticeable, not very desirable	Neutral, noticeable	Desirable, perceptible	Very desirable, very strong
Appearance	Unattractive, visible defects, uneven surface	Imperfect, uneven, reduced aesthetics	Acceptable, uniform shape and structure	Aesthetic, uniform, appetizing	Extremely attractive, smooth surface, natural color
Taste	Unpleasant, very faint	Hardly noticeable, not very desirable	Neutral, noticeable	Desirable, perceptible	Very desirable, very strong
Consistency	Unpleasant, too dry/wet, lumpy or hard	Uneven, too soft or compact	Moderate, slightly soft or compact	Uniform, well-compact, creamy	Perfect, silky, smooth, slightly moist
Color	Patchy, pale, discolored, unappetizing	Slightly uneven or pale	Acceptable, with slight shades	Uniform, natural, typical of pâté	Intense, uniform, attractive

## Data Availability

The original contributions presented in the study are included in the article; further inquiries can be directed to the corresponding author.
